# Stimulation of Proliferation and Migration of Mouse Macrophages by Type B CpG-ODNs Is F-Spondin and IL-1Ra Dependent

**DOI:** 10.1371/journal.pone.0128926

**Published:** 2015-06-04

**Authors:** Tai-An Chen, Chiao-Chun Liao, Yung-Chih Cheng, Yen-Po Chen, Yi-Fan Hsu, Chi-Ming Liang, Shu-Mei Liang

**Affiliations:** 1 Agricultural Biotechnology Research Center, Academia Sinica, Taipei, Taiwan; 2 Genomics Research Center, Academia Sinica, Taipei, Taiwan; Rutgers—New Jersey Medical School, UNITED STATES

## Abstract

Macrophage proliferation and migration are important for many facets of immune response. Here we showed that stimulation of macrophages with type B CpG oligodeoxynucleotides (CpG-B ODNs) such as CpG-ODN 1668 increased the production of anti-inflammatory cytokine interleukin 1 receptor antagonist (IL-1Ra) in a TLR9- and MyD88-dependent manner. The CpG-B ODNs-induced IL-1Ra increased macrophage migration and promoted macrophage proliferation by down-regulating the expression of a cell cycle negative regulator, p27 to increase cell population in the S phase. The induction of IL-1Ra by CpG-B ODNs was F-spondin dependent. Knockdown of F-spondin and IL-1Ra decreased CpG-B ODNs-induced macrophage migration whereas overexpression of IL-1Ra increased migration of those cells. These findings demonstrated novel roles for F-spondin and IL-1Ra in CpG-B ODNs-mediated cell proliferation and migration of macrophages.

## Introduction

Unmethylated CpG dinucleotides present in bacterial DNA are recognized by pattern recognition receptor Toll-like receptor 9 (TLR9) which triggers downstream signaling to activate target genes [1,2]. Like the unmethylated CpG motif in bacterial DNAs, synthetic oligodeoxynucleotides bearing CpG motifs (CpG ODNs) can also bind to TLR9 and activate immune responses [3]. CpG ODNs can be classified into 4 classes: type A (CpG-A ODNs), type B (CpG-B ODNs), type C, and type P [4]. CpG-A ODNs activate NK cells and stimulate plasmacytoid dendritic cells (pDCs) and macrophages to produce high levels of interferon-α [5,6]. In contrast, CpG-B ODNs primarily stimulate B cell proliferation and secretion of immunoglobulins IL-6 and IL-10. CpG-B ODNs also induce maturation and activation of pDCs and macrophages [6,7], and protect B cells, pDCs and macrophages from apoptosis [8–10]. In addition, CpG-B ODNs have been shown to induce macrophage migration by NF-κB activation and MMP-9 expression [11].

F-spondin is a secreted adhesion molecule that was originally isolated from the embryonic floor plate of vertebrates [12,13], and is known to regulate the development of the nervous system [14,15]. We previously demonstrated that F-spondin prevents the death of murine neuroblastoma cells induced by serum-starvation and cytotoxic Aβ_1~42_ peptide through maintaining IL-6 expression [16]. It has also been reported that F-spondin regulates integrin-dependent migration and adhesion of hermaphroditic specific neurons *in vivo* [14]. These studies indicate that F-spondin is critical for cytokine production and migration of neural cells. Using proteomics approaches, we previously found that CpG-B ODN treatment up-regulates F-spondin in swine peripheral blood mononuclear cells [17]. Nevertheless, the role of F-spondin in immune cells is not well understood.

Interleukin-1 receptor antagonist (IL-1Ra) binds to IL-1 type 1 receptor to block IL-1 signaling and elicits anti-inflammatory responses [18]. In addition to modulation of inflammation, IL-1Ra also has an effect on cell proliferation. Studies on endothelial cells have shown that the intracellular isoform of IL-1Ra promotes proliferation of these cells and its expression may contribute to re-endothelialization after vascular injury [19,20]. A higher proliferation rate of hepatocytes was also observed in mice treated with recombinant human IL-1Ra [21]. It is not clear, however, whether F-spondin and/or IL-1Ra play any role in CpG-ODN-driven immune responses.

In this study, we demonstrated that CpG-B ODNs, but not CpG-A ODNs, induced IL-1Ra expression in RAW 264.7 cells in a TLR9- and MyD88-dependent manner. The up-regulation of IL-1Ra in response to CpG-B ODN treatment was F-spondin dependent. The F-spondin/IL-1Ra signaling triggered by CpG-B ODN increased not only migration but also proliferation of macrophages. The effects of CpG-B ODN on the proliferation of macrophages were further explored by analyzing the cell cycle progression in the presence or absence of IL-1Ra overexpression.

## Materials and Methods

### Reagents

CpG ODN1668 (5′-TCC ATG ACG TTC CTG ATG CT-3′), GpC ODN1668 (5′-TCC ATG AGC TTC CTG ATG CT-3′), CpG-ODN2006 (5′-TCG TCG TTT TGT CGT TTT GTC GTT-3′) and Is ODN 6 (5′-GGG CAA CGT TCG ACG-3′) were synthesized with a phosphorothioate backbone at MDBio (Taipei, Taiwan). CpG ODN 1585 (5′-GGG GTC AAC GTT GAG GGG GG-3′) and GpC ODN 1585 (5′-GGG GTC AAG CTT GAG GGG GG-3′) were purchased from InvivoGen (San Diego, CA). Lipofectamine 2000 and Amaxa cell line nucleofector kit V were obtained from Invitrogen (Life Technologies, Taiwan) and Lonza (Allendale, NJ), respectively. Chloroquine and was purchased from Sigma (St. Louis, MO). Recombinant F-spondin proteins (rF-spondin), mouse IL-1Ra ELISA kit and anti-IL-1Ra antibody were purchased from R&D Systems (Minneapolis, MN). Anti-phospho CDK2 and anti-p27kip1 antibodies were obtained from Cell Signaling Technology (Danvers, MA). Anti-CDK2 and anti-actin antibodies as well as siRNAs for *F-spondin* and *IL-1Ra* genes were obtained from Santa Cruz Biotechnology (St. Louis, MO). Anti-cyclin A antibody was purchased from GeneTex (Irvine, CA), anti-GAPDH was from Abcam (Cambridge, MA), and anti-p21cip1 was from Upstate (Millipore, Bedford, MA). Anti-F-spondin and anti-Myc antibodies were purchased from Biorbyt (Cambridge, UK) and Abgent (San Diego, CA), respectively.

### Cell culture

Cell lines used in this study were purchased from American Type Culture Collection unless otherwise specified. Murine macrophage cell lines RAW264.7 and J774A.1 were maintained in Dulbecco’s modified Eagle’s medium (DMEM high and low glucose, respectively; Biowest, France) supplemented with 10% heat-inactivated fetal bovine serum (FBS; Gibco, Life Technologies), 2 mM L-glutamine, 100 U/ml penicillin and 100 μg/ml streptomycin in a humidified incubator at 37°C under 5% CO_2_. THP1 cells were maintained in RPMI-1640 (Biowest) with the supplements described above and THP1-XBlue-defMyD cells were purchased from InvivoGen and cultured according to the manufacturer’s instructions.

### Isolation of bone marrow derived macrophages

Bone marrow derived macrophages (BMMs) were harvested from BALB/c mice as described previously [22]. In brief, bone marrow was collected from femurs and tibias of mice, and the cells in bone marrow were treated with red blood cells (RBCs) lysis buffer to remove RBCs. After washing three times with RPMI-1640 medium, the cells were seeded onto 10 cm non-tissue culture dishes and cultured in RPMI-1640 medium containing 10% FBS, 20 mM 4-(2-hydroxyethyl) piperazine-1-ethanesulfonic acid (HEPES), 50 μM β-mercaptoethanol, 100 units/ml penicillin, 100 μg/ml streptomycin and 20 ng/ml granulocyte macrophage colony-stimulating factor (R&D Systems). Five milliliters of fresh culture medium was added to each dish every two days. Six days after bone marrow isolation, the medium was removed, and the cells attached to the plates were collected for further experiments.

### RNA isolation and RT-PCR

Total RNA was extracted from cells using RNeasy kit (Qiagen, Germany) according to the manufacturer’s protocol. Amplification of specific target cDNA was performed using one microgram of total RNA and a one-step RT-PCR kit (GeneMark, Taiwan) which is designed to conduct the reverse transcription and PCR amplification in one tube from either total RNA or mRNA. The primers used were: forward 5′-ATG GAA ATC TGC TGG GGA CC-3′ and reverse 5′-CTA TTG GTC TTC CTG GAA GT-3′ for mouse IL-1Ra; forward 5′-GCC TCC GCA GTC ACC TAA TC-3′ and reverse 5′-CGC TGT CTG AGC GGA TGA AG-3′ for human IL-1Ra; forward 5′-AGC TTT CTC AGA TGA GAC CC-3′ and reverse 5′-CGT TTC TGT ACA ATG CTG GC-3′ for F-spondin; and forward 5′-TGG AAT CCT GTG GCA TCC ATG AAA C-3′ and reverse 5′-TAA AAC GCA GCT CAG TAA CAG TCC G-3′ for β-actin. PCR products were subjected to electrophoresis in a 1–2% agarose gel prepared with nucleic acid stain SafeView (Applied Biological Materials, Richmond, BC).

### Plasmid construction

For mouse F-spondin silencing, a target sequence 5′-TCC TAC TTC AGA GGT TTC ACG TTA A-3′ was used, and the control sequence was 5′-TCC TTC GAC GGA TTT CAC TGT ATA A-3′. The sequences were individually cloned between the BamHI and HindIII sites downstream of the U6 promoter in the pSilencer 2.1-U6 neo plasmid (Ambion, Austin, TX). To create an expression construct to overexpress F-spondin, the full-length cDNA clone of F-spondin gene in pBluescript was purchased from Open Biosystems (Thermo Scientific, IL), and the F-spondin expression construct was generated using pcDNA3.1/myc-His (-) vector (Invitrogen). Briefly, specific primers with HindIII and XhoI restriction enzyme sites were used to amplify full-length human F-spondin ORF, and the PCR product was subsequently cloned into PCRII vector by Topo TA cloning (Invitrogen). Full-length human F-spondin with Hind III and Xho I sites in the PCRII vector was then released by restriction enzymes, purified, and subcloned into the pcDNA3.1/myc-His (-) vector. The IL-1Ra plasmid was constructed by introducing the BamHI and HindIII sites at the front and back ends of the full length mouse IL-1Ra (NC_000068.7). The following primers were used to generate PCR products: 5′-GGA TCC ATG GAA ATC TGC TGG GGA CC -3′ (forward primer with BamHI site) and 5′-AAG CTT TTG GTC TTC CTG GAA GTA GA -3′ (reverse primer with HindIII site). The sequence of the PCR product was verified by a core lab at the Institute of Biomedical Sciences, Academia Sinica, and subsequently ligated into pGEM-T Easy vector (Promega, Madison, WI) to generate the intermediate vector. The cDNA fragment encoding mIL-1Ra was then released from the intermediate vector using restriction enzymes BamHI and HindIII, and ligated into pcDNA3.1/myc-His (-) vector.

### Transfection

The stable cell lines used in this study were established by transfection of RAW264.7 cells with F-spondin shRNA, IL-1Ra plasmid or their controls using Lipofectamine 2000 (Invitrogen). The transfected cells were further selected by G-418 sulfate (Geneticin, Gibco) and ultimately maintained in DMEM containing 1 mg/ml Geneticin. For transient transfections, siRNAs or plasmids were transfected into cells by electroporation using Amaxa cell line nucleofector kit V (for RAW264.7 cells) or Mouse Macrophage Nucleofector Kit (for mouse BMMs) according to the manufacturer’s instructions.

### Western blotting

Total proteins were extracted from cells using protein extraction reagents containing protease inhibitors (Santa Cruz), and the concentration of protein extract was determined by Bio-Rad protein assay (Bio-Rad, Hercules, CA). Equivalent amounts of protein from each sample were resolved by SDS-PAGE and subsequently transferred onto a PVDF membrane (Millipore). The blots were blocked with TBS containing Tween 20 and 5% skim milk for 1 h and then incubated with specific primary antibodies, followed by horseradish peroxidase-conjugated secondary antibodies. Immunoreactivity was detected using chemiluminescent substrate (Millipore).

### Flow cytometric analysis

For cell cycle analysis, an equivalent number of wild type, vector stably transfected and IL-1Ra plasmid stably transfected RAW264.7 cells were plated into dishes and cultured for 48 h. Cells were harvested and washed with PBS once before being fixed in cold 70% ethanol for 1 h. Cell pellets were then collected by centrifugation, washed with PBS twice and treated with RNase A solution (Invitrogen). PI solution (Sigma) was added directly to cells in RNase A solution and the samples were analyzed by a FACSCalibur system (BD Biosciences, Bedford, MA) after incubation for 30 min at room temperature. The data were analyzed and plotted using ModFit LT software (Verity Software House, USA).

### Time-lapse microscopy

Time-lapse microscopy was performed as described previously [22]. Briefly, the movement of cells was observed by an inverted Leica Axiovert 200 light microscope equipped with a humidified chamber which was maintained at 37°C with 5% CO_2_. Serial phase-contrast images were obtained every 15 min for 22 h, and the cell trajectories and migration velocities were determined using the tracking point tool of Metamorph software (Molecular Devices, Sunnyvale, CA). The average of cell migration velocities was defined as the average of 20 subsequent cell centroid displacements/one time interval between two images.

### Statistical analysis

Statistically significant difference between two groups of data was determined using a 2-tailed Student's t test. All data analyses were performed with GraphPad Prism 5.0 for Microsoft Windows (GraphPad Software, La Jolla, CA). *P* values less than 0.05 were considered statistically significant. *P < 0.05; **P < 0.01; ***P < 0.001.

## Results

### CpG-B ODNs increase IL-1Ra expression in a TLR9- and MyD88-dependent manner

**Fig 1:**
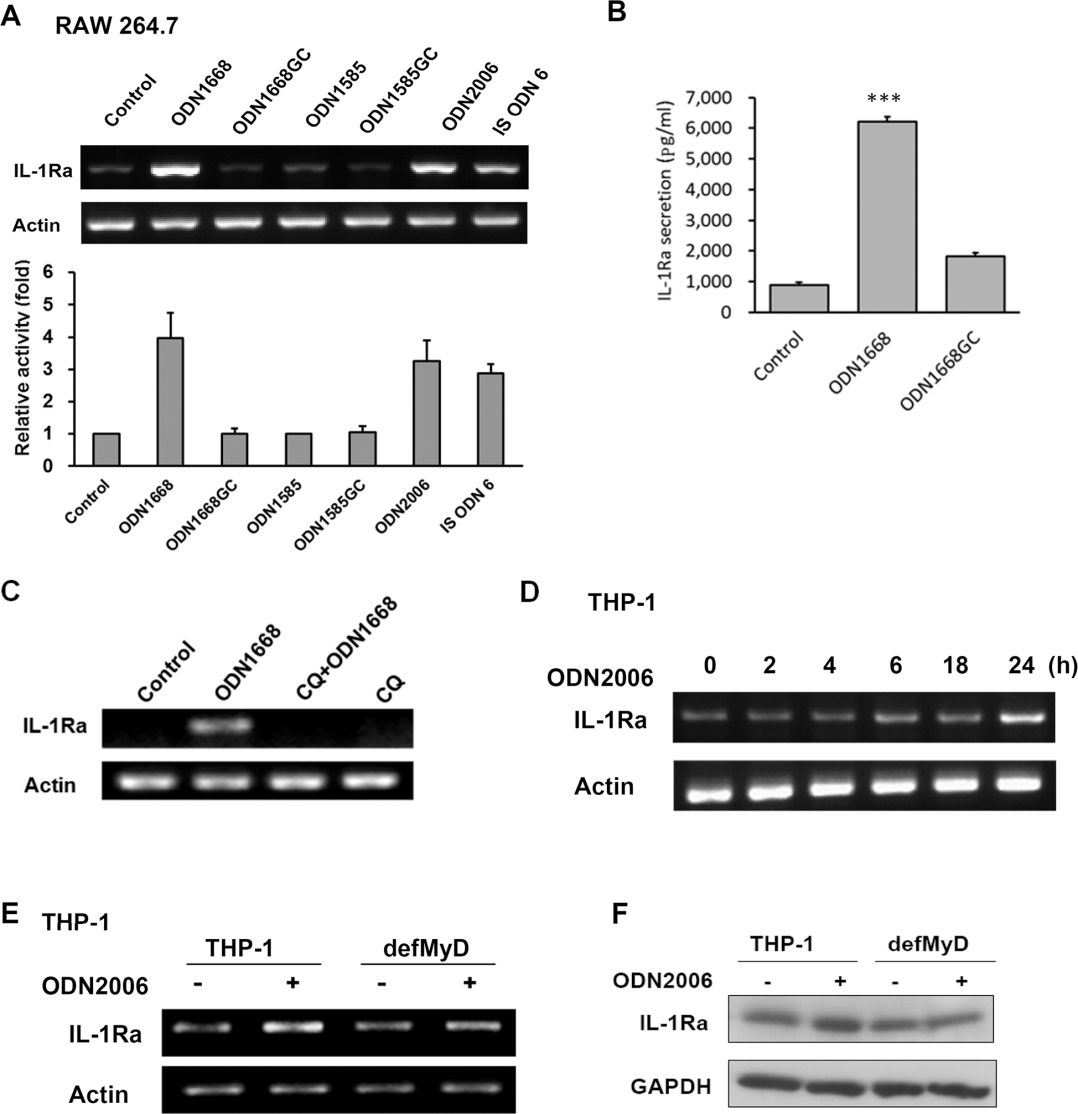
Type B CpG ODNs increase IL-1Ra expression in a TLR9- and MyD88-dependent manner. (A) RAW264.7 cells were treated with or without 1 μM of various ODNs and their controls for 6 h and the mRNA expression of IL-1Ra was analyzed by RT-PCR. The gel shown is the representative of three independent experiments (means ± SEM). (B) IL-1Ra production of cells treated with/without 6 μM ODN1668 for 24 h was measured by ELISA; ***, *P* < 0.001. (C) Cells were treated with or without 20 μM chloroquine in complete medium for 1 h prior to ODN treatment (1 μM). Six hours later, cells were harvested for RT-PCR analysis. (D) THP-1 cells were treated with 2 μM ODN2006 for the times indicated and assayed for IL-1Ra mRNA expression. (E-F) THP-1 and MyD88 deficient THP-1 (defMyD) stable cell lines were treated with or without 2 μM ODN2006 for 24 h. The expression of IL-1Ra was analyzed by both RT-PCR (E) and western blotting (F).

To determine the effect of CpG ODNs on IL-1Ra expression in macrophages, we first compared the response of mouse macrophage cell line RAW264.7 to CpG-A or CpG-B ODNs. The mRNA level of IL-1Ra in cells treated with CpG-B ODNs, including ODN1668, ODN2006, and ISODN6, was increased compared with that in cells treated with GpC ODN (negative control) (Fig 1A). Cells treated with CpG-A ODNs such as ODN1585, on the other hand, did not show increased IL-1Ra expression (Fig 1A). In line with the mRNA level, ODN1668 treatment increased IL-1Ra protein secretion of RAW264.7 cells as demonstrated by ELISA (Fig 1B). Since CpG ODNs are well-known ligands for TLR9 [1,2], we then examined whether TLR9 is essential for CpG-B ODNs-induced IL-1Ra expression. When RAW264.7 cells were pre-treated with chloroquine, an inhibitor of endocytic TLRs known to abolish TLR signaling [23], subsequent treatment with ODN1668 did not cause an increase in IL-1Ra expression in these cells (Fig 1C). Myeloid differentiation protein-88 (MyD88) is a key adapter in TLR signaling [1], therefore, we further investigated whether MyD88 is involved in the induction of IL-1Ra by CpG-B ODNs. Human monocytic THP-1 and a stable mutant cell line that is deficient in MyD88 activity were used for this purpose. IL-1Ra expression was first found to increase gradually within 24 h of CpG-B ODN treatment in wild-type THP-1 cells (Fig 1D). Both RT-PCR and western blotting analyses showed that CpG-B ODNs-induced IL-1Ra expression was attenuated in MyD88 deficient THP-1 cells (Fig 1E and 1F). Taken together, these results indicate that CpG-B but not CpG-A ODNs increase the expression of IL-1Ra via a TLR9/MyD88 signaling pathway.

### F-spondin mediates the up-regulation of IL-1Ra by CpG-B ODNs

**Fig 2:**
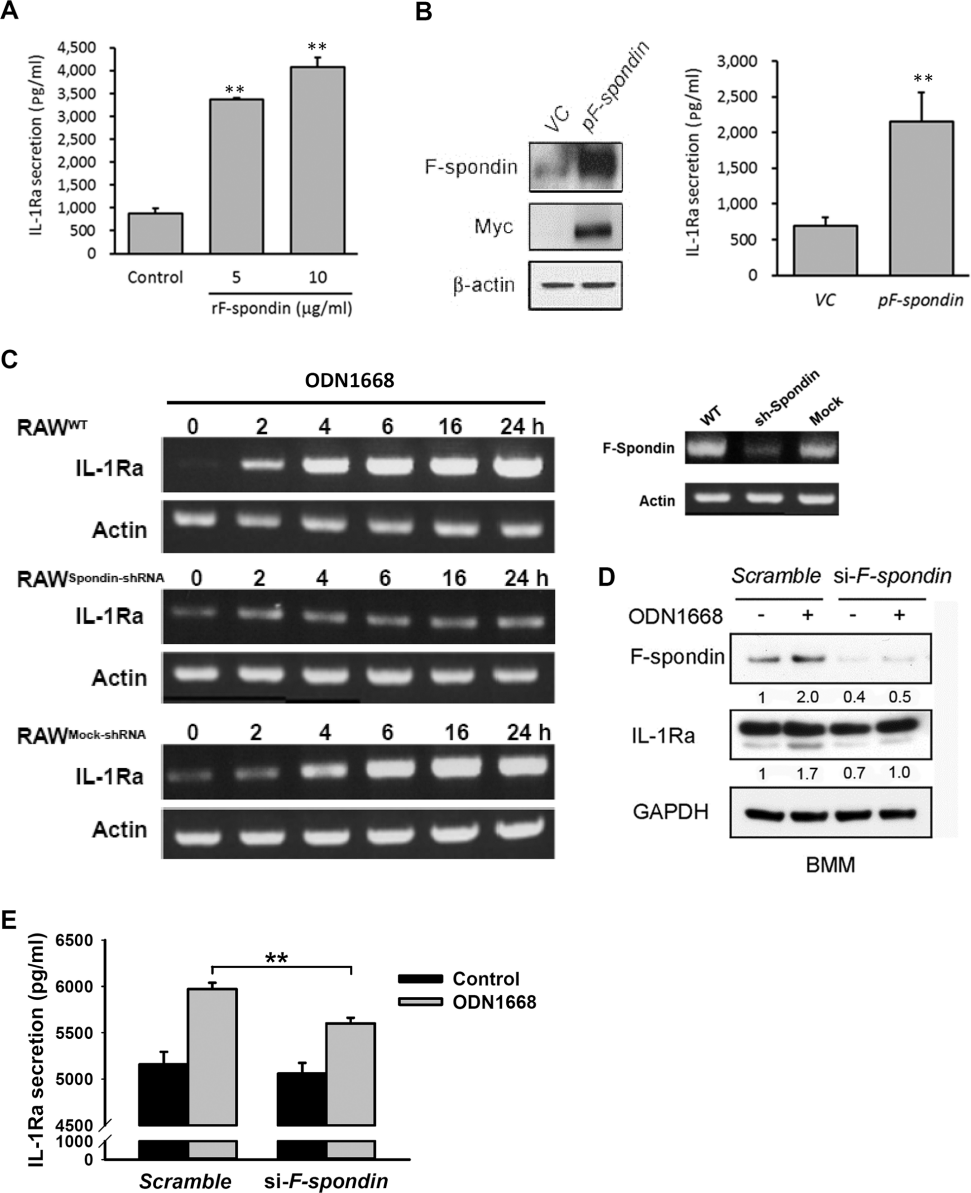
ODN1668 increases IL-1Ra expression via F-spondin. (A) RAW264.7 cells were stimulated with recombinant F-spondin proteins (rF-spondin) for 24 h and IL-1Ra production was measured by ELISA; **, *P* < 0.01. (B) RAW264.7 cells were transfected with either Myc-tagged F-spondin plasmid (pF-spondin) or a control vector (VC), and incubated for 24h. The success of transfection was verified by western blotting analysis of F-spondin and Myc expression. The IL-1Ra production was measured by ELISA 48h after transfection; **, *P* < 0.01. (C) Effect of F-spondin knockdown on ODN1668-induced IL-1Ra expression. Knockdown of F-spondin was verified by RT-PCR analysis. Wild-type RAW264.7 cells (RAW^WT^) and RAW264.7 cells stably transfected with F-spondin shRNA (RAW^spondin-shRNA^) or scrambled shRNA (RAW^Mock-shRNA^) were incubated with or without 1 μM ODN1668 for the indicated times. RT-PCR was then performed to analyze IL-1Ra expression. β-actin was used as a loading control. (D) Bone marrow derived macrophages were transfected with scrambled siRNA or siRNA for *F-spondin* gene. After cells were attached to the plate, media were removed and fresh media containing 6 μM ODN1668 were supplied. The expressions of F-spondin and IL-1Ra were examined after 1d treatment. Similar results were obtained in two independent experiments. (E) The IL-1Ra production of cells in (D) was measured by ELISA; **, *P* < 0.01. Data shown are average of three independent experiments.

We previously found that F-spondin plays a role in regulating cytokine production [16]. To examine whether or not F-spondin regulates IL-1Ra production, we first treated RAW264.7 cells with recombinant F-spondin proteins (rF-spondin), and showed that treatment with rF-spondin increased IL-1Ra production (Fig 2A). Similar effect was demonstrated by over-expression of F-spondin in RAW264.7 cells (Fig 2B). To further examine whether F-spondin is involved in CpG-B ODNs-mediated IL-1Ra expression, a control stable RAW264.7 cell line transfected with mock shRNA (RAW^Mock-shRNA^) and a stable RAW264.7 cell line transfected with F-spondin shRNA (RAW^spondin-shRNA^) were generated, and the silencing effect of F-spondin shRNA was verified by RT-PCR (Fig 2C, right panel). Within 24 h of ODN1668 treatment, a time-dependent increase in IL-1Ra mRNA level was observed in RAW264.7 cells (RAW^WT^) and RAW^Mock-shRNA^ cells, but not in RAW^spondin-shRNA^ cells (Fig 2C, left panel), suggesting that the up-regulation of IL-1Ra induced by ODN1668 is mediated by F-spondin. Next, we used mouse bone marrow derived macrophage (BMMs) to further confirm the involvement of F-spondin in CpG-B ODNs-mediated IL-1Ra expression in primary cells. Downregulation of F-spondin in BMMs diminished the effect of ODN1668 on expression (Fig 2D) and secretion (Fig 2E) of IL-1Ra. These findings confirm that F-spondin plays a role in mediating the effect of CpG-B ODNs on IL-1Ra secretion in macrophages.

### IL-1Ra increases cell proliferation via enhancing S phase of the cell cycle

**Fig 3:**
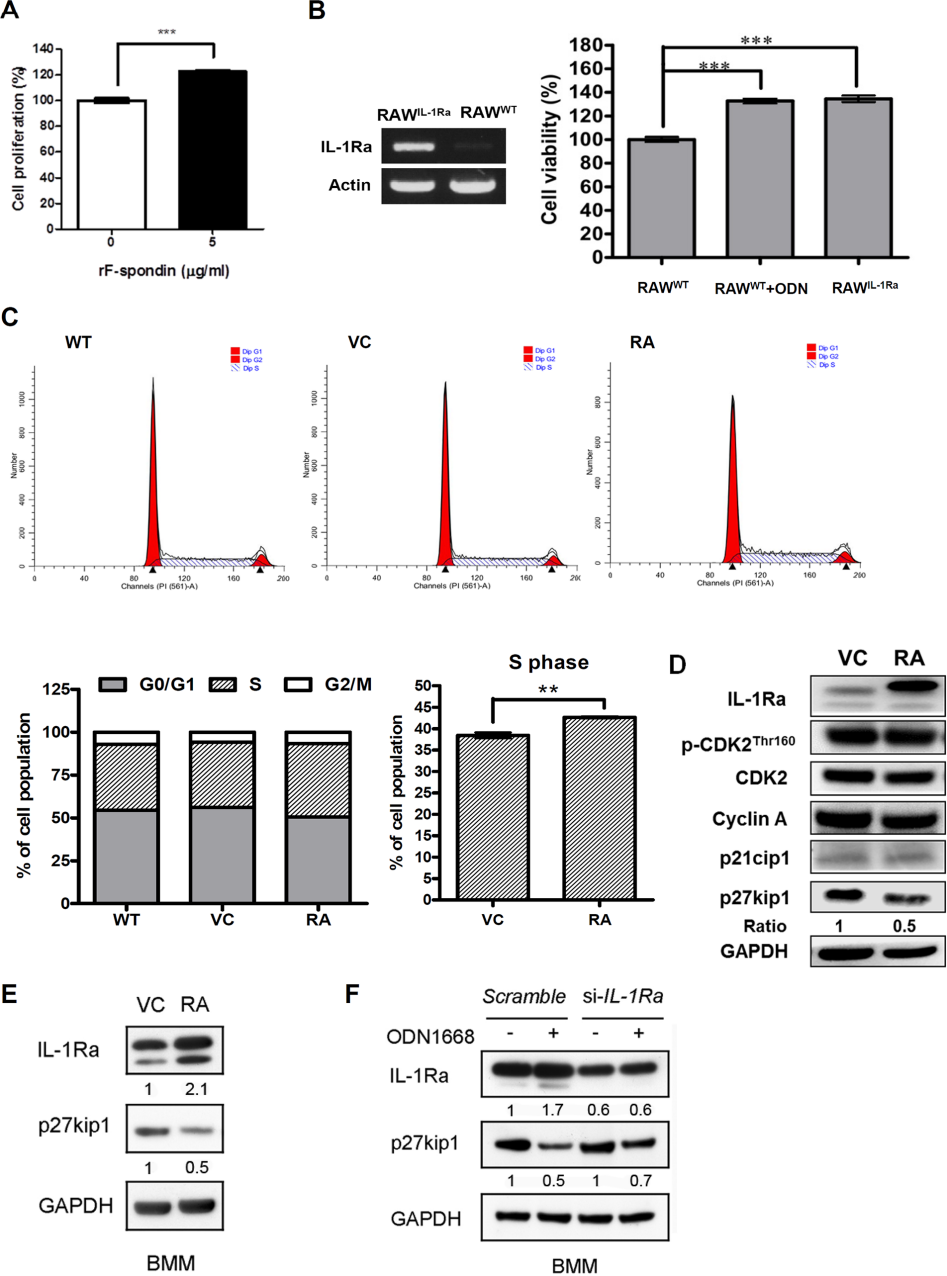
Overexpression of IL-1Ra increases RAW 264.7 cell proliferation through increasing S phase cell population. (A) RAW264.7 cells treated with or without 5 μg/ml recombinant F-spondin protein (rF-spondin) were cultured in complete media for 48 h and assayed for viability; ***, *P* < 0.001. (B) RAW264.7 cells treated with or without 1 μM ODN1668 and IL-1Ra overexpressing stable cell line (RAW^IL-1Ra^) were cultured in complete media for 48 h and assayed for viability; ***, *P* < 0.001. Overexpression of IL-1Ra in RAW^IL-1Ra^ was confirmed by RT-PCR. (C) Wild-type RAW264.7 cells (WT), RAW264.7 cells stably transfected with empty vector (VC), and RAW^IL-1Ra^ cells (RA) were plated at the same density and cultured in complete media for 48 h. Cell cycle analysis was performed by flow cytometry and the data were analyzed using ModFit software. Results shown are representative of three independent experiments; **, *P* < 0.01. (D) The expression of cell cycle-related protein in RAW^VC^ and RAW^IL-1Ra^ cells were examined by western blotting. (E) Bone marrow derived macrophages (BMMs) were transiently transfected with a control vector (VC) or IL-1Ra plasmid (RA). The expressions of IL-1Ra and p27 were examined 24h after transfection. (F) Western blotting analysis of IL-1Ra and p27 expressions in BMMs transfected with scrambled siRNA or siRNA for *IL-1Ra* gene. Cells were incubated with 6 μM ODN1668 for 24h before harvest.

IL-1Ra has been reported to modulate proliferation of endothelial cells [20]. To investigate whether the F-spondin/IL-1Ra signaling triggered by CpG-B ODN may contribute to ODN1668-induced macrophage proliferation, we first treated RAW 264.7 cells with rF-spondin. Cells treated with rF-spondin showed an approximately 20% increase in proliferation (Fig 3A). Next, we stably transfected RAW 264.7 cells with IL-1Ra plasmid (RAW^IL-1Ra^) and compared their proliferation with that of ODN1668-treated cells. Our results revealed that like ODN1668-treated cells, RAW^IL-1Ra^ showed a proliferation rate 30% higher than ODN1668-untreated naive cells (Fig 3B). To further examine the effect of IL-1Ra on cell cycle progression, RAW^WT^, RAW^vector control (VC)^, and RAW^IL-1Ra^ cells were subjected to flow cytometry analysis. Over-expression of IL-1Ra decreased the G_0_/G_1_ phase and increased the S phase of the cell population (Fig 3C). Western blotting analysis revealed that IL-1Ra over-expression down-regulated the expression of a cell cycle negative regulator, p27, in RAW264.7 cells (Fig 3D). These data allowed us to infer that the down-regulation of p27 induced by IL-1Ra may at least in part contribute to the shift toward S phase of the cell cycle in IL-1Ra over-expressing RAW264.7 cells. The association between IL-1Ra and p27 was further confirmed in BMMs. Transient transfection of BMMs with IL-1Ra plasmid led to a decrease in p27 expression (Fig 3E). In addition, knockdown of IL-1Ra with siRNA partially reversed the down-regulating effect of ODN1668 on p27 in BMMs (Fig 3F), providing extra evidence that IL-1Ra plays a role in ODN1668-induced macrophage proliferation via down-regulating p27.

### CpG-B ODNs increase mouse macrophage migration via F-spondin and IL-1Ra

**Fig 4:**
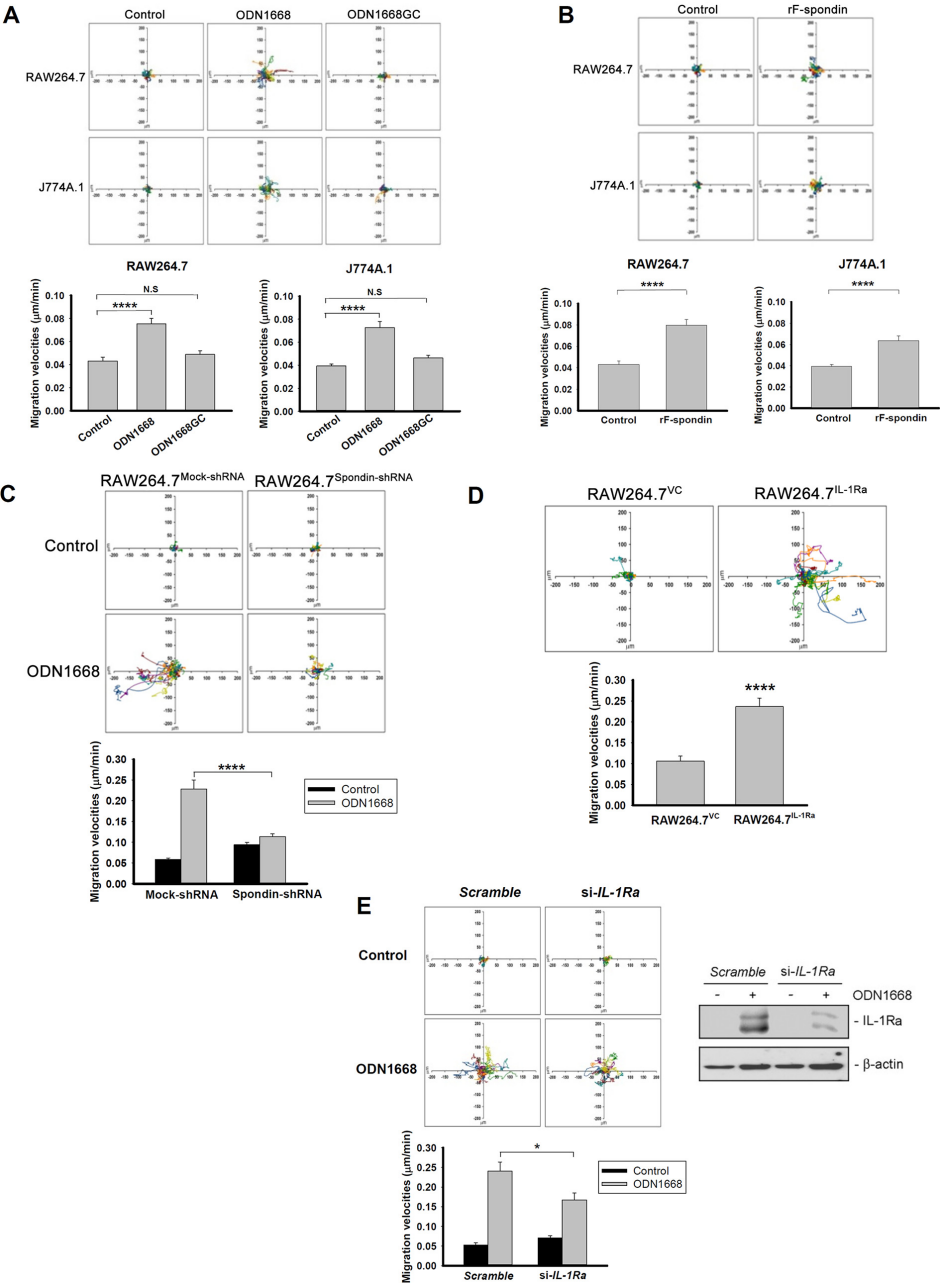
ODN1668 promotes macrophage migration via the F-spondin/IL-1Ra signaling pathway. Trajectories of cells in response to different treatments were measured by time-lapse microscopy and displayed in diagrams drawn with the initial point of each trajectory placed at the origin of the plot. Serial phase-contrast images were obtained every 15 min for 22 h by time-lapse microscopy. Trajectories (upper panel) and migration velocity (lower panel) of cells were displayed after 22 h of time-lapse. Results of migration velocities represent data of 20 cells (means ± SEM). (A) RAW264.7 and J774A.1 cells were treated with or without 6 μM ODN1668 or 6 μM GpC ODN1668 (ODN1668GC). (B) RAW264.7 and J774A.1 cells were treated with or without 2 μg/ml recombinant F-spondin protein (rF-spondin). (C) RAW^Mock-shRNA^ and RAW^Spondin-shRNA^ stable cell lines were treated with or without 6 μM ODN1668. (D) The trajectory and migration velocity of untreated RAW^VC^ and RAW^IL-1Ra^ stable cell lines were compared. (E) RAW264.7 cells were transfected with scrambled siRNA or siRNA for *IL-1Ra* gene. After cells were attached to the plate, media were removed and fresh media containing 6 μM ODN1668 were supplied. The transfection efficiency was verified by western blotting. *, *P* < 0.05, ****, *P* < 0.0001, N.S., not significant.

**Fig 5:**
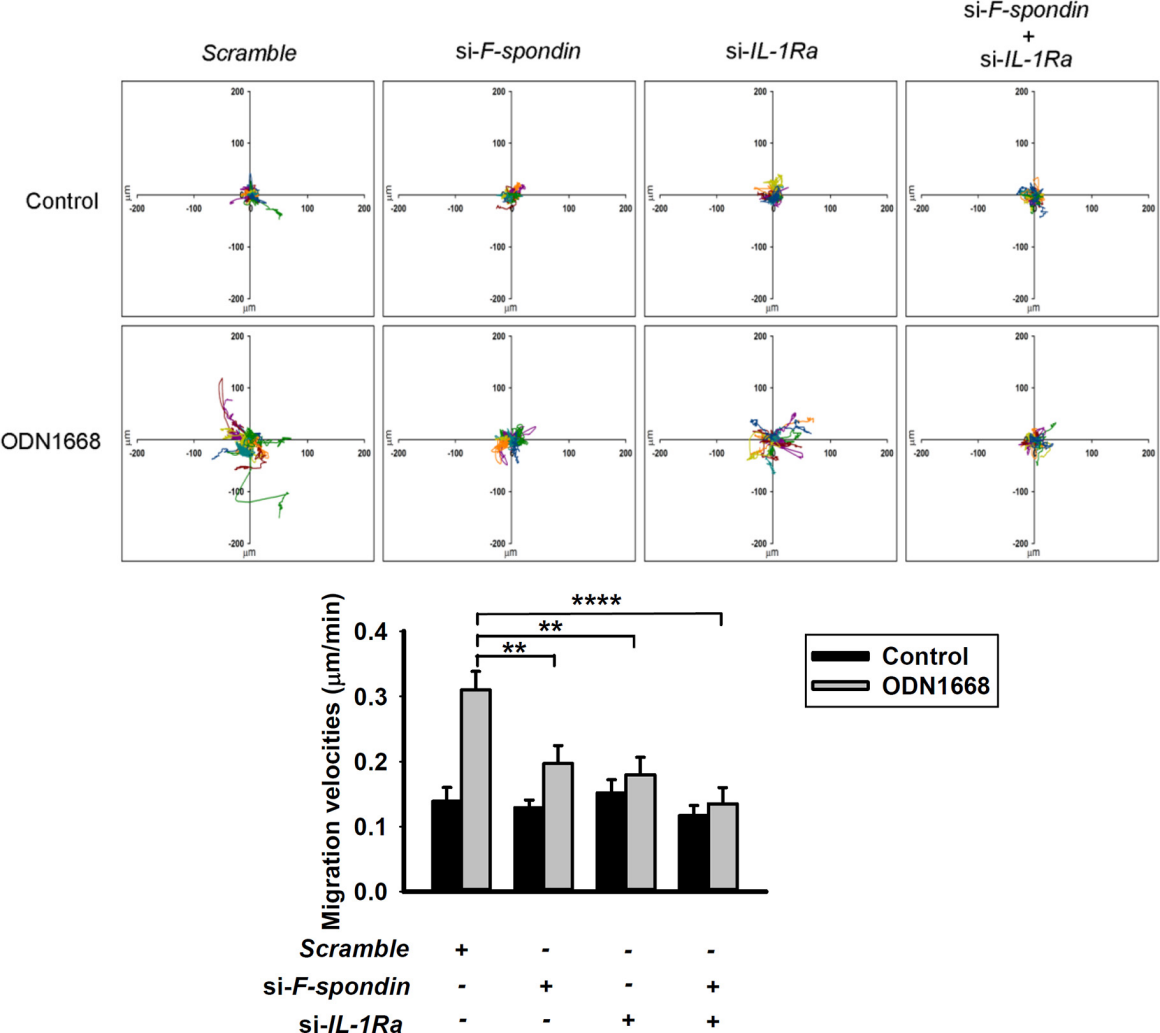
Knockdown of F-spondin and IL-1Ra diminishes the effect of ODN1668 on migration of bone marrow derived macrophages (BMMs). BMMs were transfected with scrambled siRNA or siRNA for *IL-1Ra* and/or *F-spondin* gene. After cells were attached to the plate, media were removed and fresh media containing 6 μM ODN1668 were supplied. Trajectories (upper panel) and migration velocity (lower panel) of cells were displayed after 22 h of time-lapse as described in the Materials & Methods section. **, *P* < 0.01, ****, *P* < 0.0001.

A CpG-B ODN has previously been reported to induce RAW264.7 cell migration by a transwell migration assay system [11]. By monitoring the trajectories of cells with time-lapse microscopy, a method that we have successfully used to evaluate macrophage migration [22], we also demonstrated that the dispersive areas of the trajectories were markedly larger in RAW264.7 cells treated with CpG ODN1668 than those of untreated cells or cells treated with negative control, i.e., GpC ODN1668 (Fig 4A, upper panel). In addition, ODN1668-treated cells exhibited higher migration velocities than control cells (Fig 4A, lower panel). These effects were also observed in another mouse macrophage cell line, J774A.1 (Fig 4A). F-spondin has been implicated in migration of non-immune cells [14]. To determine whether F-spondin also regulates migration of immune cells, we treated RAW264.7 and J774A.1 cells with rF-spondin and found that like ODN1668, rF-spondin increased migration of both cell lines (Fig 4B). Furthermore, knockdown of F-spondin greatly diminished the effect of ODN1668 on RAW264.7 cell migration (Fig 4C). Since we have demonstrated that ODN1668 increased IL-1Ra expression via F-spondin (Fig 2), we then further investigated the effect of IL-1Ra on macrophage migration by over-expression or knockdown of *IL-1Ra* gene in RAW264.7 cells. By comparing the migration capability of RAW^VC^ and RAW^IL-1Ra^ cells, it was found that over-expression of IL-1Ra significantly enhanced the migration of RAW264.7 cells (Fig 4D); on the other hand, knockdown of IL-1Ra with siRNA decreased the effect of ODN1668 on RAW264.7 cell migration (Fig 4E). Similar results were demonstrated in BMMs. Knockdown of F-spondin, IL-1Ra, or both significantly lessened ODN1668-induced migration of BMMs (Fig 5). Collectively, these data indicate that ODN1668 enhances macrophage migration by up-regulation of F-spondin and IL-1Ra.

## Discussion

IL-1Ra has been shown to stimulate proliferation of non-immune cells, such as endothelial cells and hepatocytes [19–21]. Although CpG ODNs are well-known immune stimulants, little has been reported about the interplay between CpG ODNs and IL-1Ra in immune cells. In this study, we found that CpG-B ODNs-induced proliferation and migration of macrophages was associated with up-regulation of IL-1Ra. Our results showed that CpG-B ODNs, but not CpG-A ODNs, significantly increased IL-1Ra mRNA expression and secretion in RAW264.7 macrophage cells (Fig 1A and 1B). These results are consistent with other studies showing that different CpG ODN classes induce distinct cytokine gene expression patterns [24]. In addition, in accordance with the studies of IL-1Ra on non-immune cells, our results demonstrated that IL-1Ra increases proliferation of macrophages (Fig 3).

Along with IL-1Ra, we also analyzed the mRNA expression of IL-1α and IL-1β in CpG-B ODN-treated RAW264.7 macrophage cells. Time-course analysis revealed that the up-regulation of IL-1Ra induced by ODN1668 persisted for 48 hours (S1 Fig). Although ODN1668 also up-regulated the expression of IL-1α and IL-1β, in contrast to IL-1Ra, the effect peaked at 6 hours post-treatment, followed by a plunge afterwards. The long-lasting effect of CpG-B ODN on IL-1Ra expression indicates that IL-1Ra may play a more important role than IL-1α and IL-1β in sustaining the biological activities of macrophages after CpG-B ODN stimulation. In accordance with a study showing that CpG-B ODN is a potent inducer of pro-inflammatory cytokines for monkey PBMCs [24], ODN1668 induced IL-6 secretion and TNFα expression in RAW264.7 cells (S2A and S2B Fig). It is of interest to note that ODN1668 also stimulated the production of anti-inflammatory cytokine IL-10 in RAW264.7 cells (S2C Fig), but failed to stimulate IL-4 (data not shown). The bi-phasic stimulatory effect of CpG-B ODN on pro- and anti-inflammatory cytokines suggests a complex system of cytokine responses to CpG-B ODN, which might involve negative-feedback mechanisms [25]. In addition, our result on NO production is also consistent with literature that CpG-B ODNs stimulate NO production in mouse macrophages (S2D Fig) [26]. Interestingly, we also found that ODN1668 decreased arginase-1 expression in RAW264.7 cells (S2E Fig), which is similar to the effect of CpG ODN on monocytic myeloid derived suppressor cells [27]. It has been reported that inhibition of arginase prevents inflammation [28]. Whether there is a correlation between arginase and IL-1Ra in the regulation of inflammation may be worthy of investigation.

The extracellular matrix-associated protein F-spondin has been implicated in cellular protection and migration of neural cells [16,29,30]. Whether it has similar function in immune cells has not been extensively investigated. Our results shown in Figs 1–3 suggest that the up-regulation of an F-spondin/IL-1Ra signaling pathway also contributes to the pro-proliferative effect of CpG-B ODNs in macrophages. To the best of our knowledge, this is the first report to show that F-spondin is related to macrophage proliferation.

In addition to macrophage proliferation, we herein provide evidence that an F-spondin/IL-1Ra signaling pathway induced by CpG-B ODNs leads to enhancement of macrophage migration. Treatment with rF-spondin or over-expression of IL-1Ra increased the dispersive capability and migration velocity of macrophages (Fig 4B and 4D), and knockdown of F-spondin attenuated ODN1668-induced cell migration (Fig 4C). We thus demonstrated that F-spondin acted as a positive regulator of macrophage migration in response to CpG-B ODN treatment. In contrast to our findings, a recent study showed that F-spondin down-regulated macrophage colony stimulating factor (M-CSF)-induced migration of osteoclastic precursors, RAW264.7 cells [31]. We speculate that F-spondin might have opposing function in different biological environments, such as in the presence of M-CSF or CpG-B ODNs. It has been reported that the proteolytic processing of F-spondin generates two functionally opposing fragments in the guidance of neuron extension [32]. Whether similar scenario occurs in macrophages awaits further investigation.

The results shown in Fig 1C–1F indicate that the up-regulation of IL-1Ra by CpG-B ODNs is TLR9- and MyD88-dependent. Since TLR9 is a well-known receptor for CpG ODNs [1–3], these results are not surprising. Nevertheless, the results presented here reveal that, in addition to the unconventional signaling pathways initiated from other possible receptors as proposed by previous studies [33,34], the traditional receptor TLR9 is also important for the effect of CpG ODNs on macrophage proliferation and migration, and is essential for IL-1Ra-mediated macrophage proliferation and migration in response to CpG ODNs.

In conclusion, we have defined new signalling pathways by which CpG-B ODNs elicit proliferation and migration of macrophages. CpG-B ODNs up-regulate the expression of F-spondin and downstream IL-1Ra to promote macrophage proliferation and migration. We have thus reported for the first time that F-spondin and IL-1Ra play a role in macrophage proliferation and migration, respectively. These findings increase our understanding of CpG ODNs-mediated proliferation and migration of macrophages.

## Supporting Information

S1 Fig Time-course analysis of IL-1Ra, IL-1α, and IL-1β expression in response to CpG ODN treatment. RAW264.7 cells were treated with or without 1 μM ODN1668 for the indicated times, and the mRNA expressions of IL-1Ra, IL-1α, and IL-1β were analyzed by RT-PCR.
(TIF)

S2 Fig Analysis of cytokine and nitric oxide (NO) production as well as arginase 1 expression in CpG ODN-treated macrophages. RAW264.7 cells were treated 1α M CpG ODN1668 or GpC ODN1668 for 24h, and the production of IL-6, IL-10, and NO was analyzed by ELISA (A, C-D). Gene expressions of TNFα and arginase 1 were analyzed by qPCR (B, E).
(TIF)

S1 Text Supporting materials and methods. (DOCX)
