# Supplementary figures and images for: Stimulation of Proliferation and Migration of Mouse Macrophages by Type B CpG-ODNs Is F-Spondin and IL-1Ra Dependent
Source: PLoS One. 2015 Jun 4;10(6):e0128926. doi: 10.1371/journal.pone.0128926 (PMC4456401; doi:10.1371/journal.pone.0128926)

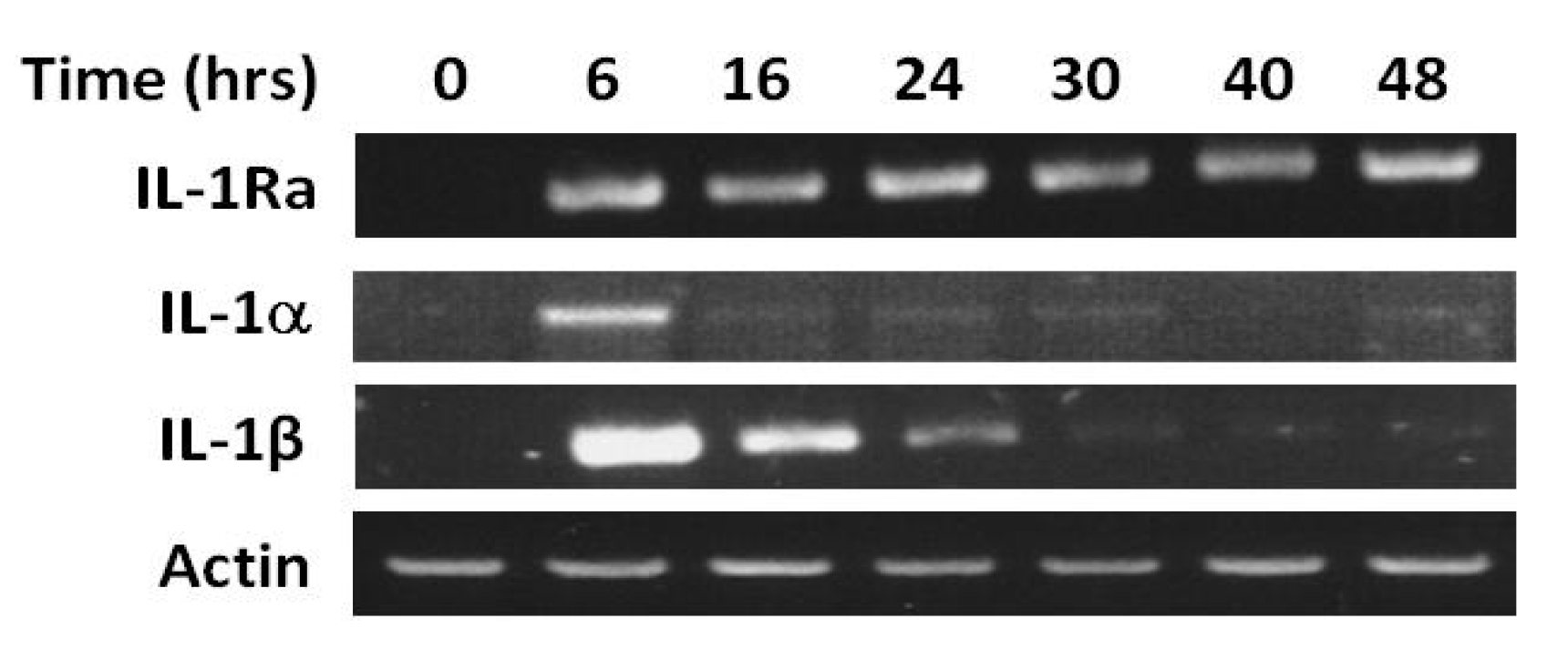

Supplement: S1 Fig — RAW264.7 cells were treated with or without 1 μM ODN1668 for the indicated times, and the mRNA expressions of IL-1Ra, IL-1α, and IL-1β were analyzed by RT-PCR. (TIF) [file pone.0128926.s001.tif]

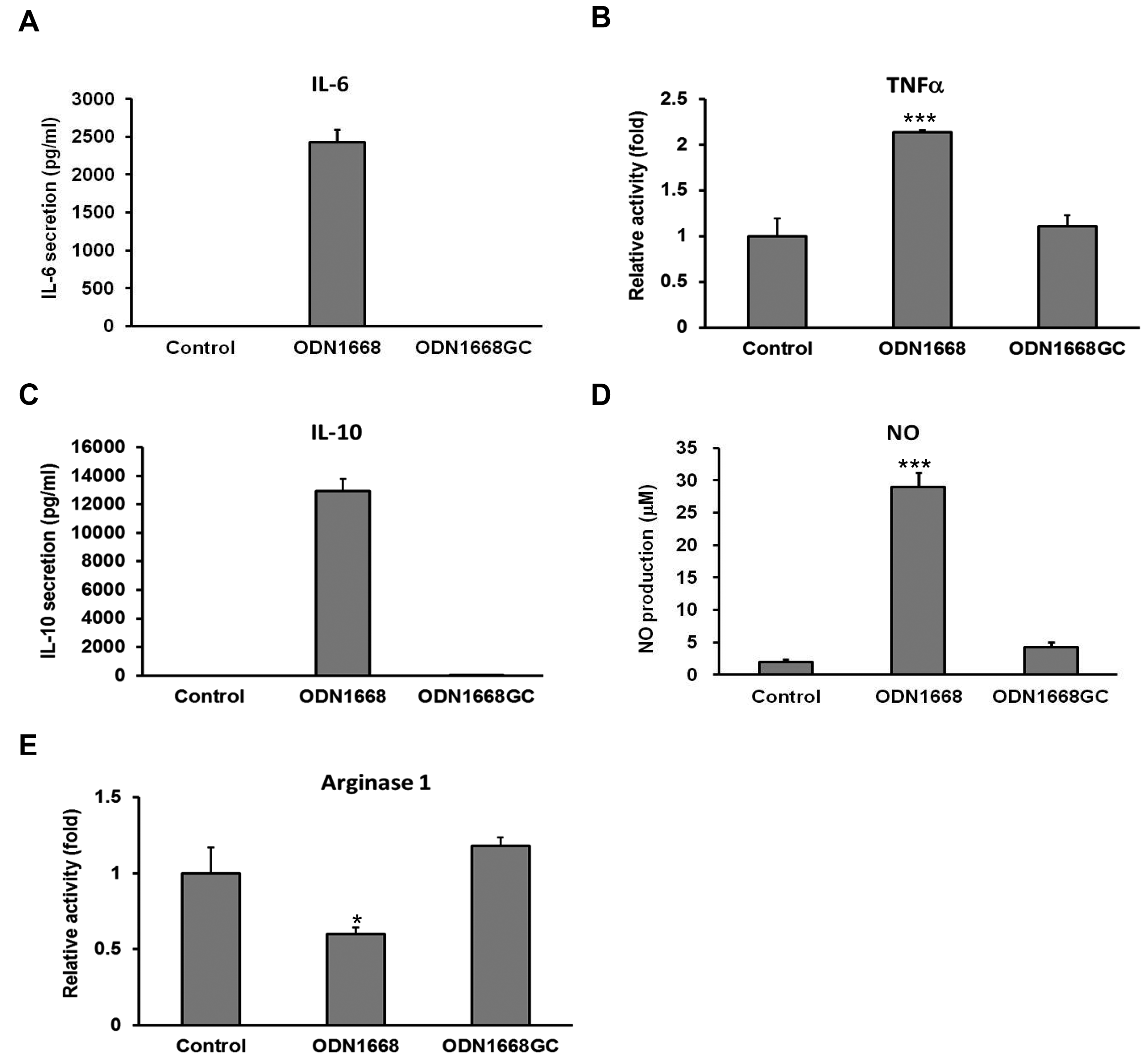

Supplement: S2 Fig — RAW264.7 cells were treated 1α M CpG ODN1668 or GpC ODN1668 for 24h, and the production of IL-6, IL-10, and NO was analyzed by ELISA (A, C-D). Gene expressions of TNFα and arginase 1 were analyzed by qPCR (B, E). (TIF) [file pone.0128926.s002.tif]
