# Supplementary material for: Stimulation of Proliferation and Migration of Mouse Macrophages by Type B CpG-ODNs Is F-Spondin and IL-1Ra Dependent
Source: PLoS One. 2015 Jun 4;10(6):e0128926. doi: 10.1371/journal.pone.0128926 (PMC4456401; doi:10.1371/journal.pone.0128926)
Supplement: S1 Text — (DOCX) [file pone.0128926.s003.docx]

**Supporting Information**

**Materials and Methods**

**PCR/qPCR**

PCR amplification of IL-1α and IL-1β were performed with primer sequence of forward 5′-ATGGCCAAAGTTCCTGACTT-3′ and reverse 5′- TTATGATATCTGGAAGTCTG -3′ for mIL-1α; forward 5′- ATGGCAACTGTTCCTGAACT -3′ and reverse 5′- TTAGGAAGACACAGATTCCA -3′ for mIL-1β.

The quantitative real-time PCR (qPCR) analysis of TNFα and Arginase 1 was conducted using an Applied Biosystems 7500 Fast Real-Time PCR System (ABI, Foster City, CA) with Power SYBR^®^ Green PCR master mix (ABI). For qPCR experiments, primer sequences of forward 5′- TTCTGTCTACTGAACTTCGGGGTGATCGGTCC -3′ and reverse 5′- GTATGA GAT AGC AAA TCGGCTGACGGTGTGGG -3′ were used for mouse TNFα, and those of forward 5′- GAACACGGCAGTGGCTTTAAC-3′ and reverse 5′- TGCTAGCTCTGTCTGCTTTGC -3′ were used for mouse Arginase 1.

**ELISA**

Mouse IL-6 and NO ELISA kits were purchased from BioSource (Life Technologies, Taiwan), and mouse IL-4 and IL-10 ELISA kits were obtained from eBioscience (San Diego, CA). Cells were treated with 1μM CpG ODN1668 or GpC ODN1668 for 24h, and media were analyzed according to the manufacturer’s instructions.
